# The use of a liner under different bulk-fill resin composites: 3D GAP formation analysis by x-ray microcomputed tomography

**DOI:** 10.1590/1678-7757-2019-0042

**Published:** 2019-11-11

**Authors:** Burcu Oglakci, Magrur Kazak, Nazmiye Donmez, Evrim Eliguzeloglu Dalkilic, Safiye Selin Koymen

**Affiliations:** 1 Bezmialem Vakif University Faculty of Dentistry, Department of Restorative Dentistry, Istanbul, Turkey

**Keywords:** Bulk-fill composite, Gap formation, Liner, Resin-modified glass-ionomer cement, Micro-computed tomography, Micro-CT

## Abstract

Gap formation of composite resin restorations is a serious shortcoming in clinical practice. Polymerization shrinkage stress exceeds the tooth-restoration bond strength, and it causes bacterial infiltration within gaps between cavity walls and the restorative material. Thus, an intermediate liner application with a low elastic modulus has been advised to minimize polymerization shrinkage as well as gap formation.

**Objective:**

The purpose of this *in vitro* study was to assess gap formation volume in premolars restored with different bulk-fill composites, with and without a resin-modified glass-ionomer cement (RMGIC) liner, using x-ray micro-computed tomography (micro-CT).

**Methodology:**

Sixty extracted human maxillary premolars were divided into six groups according to bucco-palatal dimensions (n=10). Standardized Class II mesio-occluso-distal cavities were prepared. G-Premio Bond (GC Corp., Japan) was applied in the selective-etch mode. Teeth were restored with high-viscosity (Filtek Bulk Fill, 3M ESPE, USA)-FB, sonic-activated (SonicFill 2, Kerr, USA)-SF and low viscosity (Estelite Bulk Fill Flow, Tokuyama, Japan)-EB bulk-fill composites, with and without a liner (Ionoseal, Voco GmbH, Germany)-L. The specimens were subjected to 10,000 thermocycles (5-55°C) and 50,000 simulated chewing cycles (100 N). Gap formation based on the volume of black spaces at the tooth-restoration interface was quantified in mm^3^ using micro-computed tomography (SkyScan, Belgium), and analyses were performed. Data were analyzed using repeated-measures ANOVA and the Bonferroni correction test (p < 0.05).

**Results:**

The gap volume of all tested bulk-fill composites demonstrated that Group SF (1.581±0.773) had significantly higher values than Group EB (0.717±0.679). Regarding the use of a liner, a significant reduction in gap formation volume was observed only in Group SFL (0.927±0.630) compared with Group SF (1.581±0.773).

**Conclusion:**

It can be concluded that different types of bulk-fill composite resins affected gap formation volume. Low-viscosity bulk-fill composites exhibited better adaptation to cavity walls and less gap formation than did sonic-activated bulk-fill composites. The use of an RMGIC liner produced a significant reduction in gap formation volume for sonic-activated bulk-fill composites.

## Introduction

Increasing demand for esthetics and improvements in adhesive system technology has made resin composite restorations a popular choice for clinicians.^1^ However, shrinkage associated with the polymerization of materials is a serious shortcoming in clinical practice.^2^ Polymerization shrinkage stress exceeds the tooth-restoration bond strength, and it causes fluid passage and bacterial infiltration within gaps between cavity walls and the restorative material.^3^ Microleakage, which is described as clinically undetectable penetration, could lead to post-operative hypersensitivity, marginal staining, secondary caries, pulpal inflammation and necrosis.^4^

Several procedures have been developed to decrease polymerization shrinkage stress, such as modifying the chemical composition in the resin formulation, control of light irradiance, incremental layering techniques and intermediate liner application.^5^ However, no definitive method to eliminate polymerization shrinkage has been described in the literature.^6^

The incremental layering is a standard protocol used to place restorative materials in the cavity, but this technique has many disadvantages, such as placement difficulty in small cavities, increased chair time, voids and contamination risk between composite layers.^7^ Therefore, novel composite resin materials with the use of bulk-filling techniques have been placed on the market.^8^ Bulk-fill composite resins can be applied in 4-5-mm thicknesses with relative ease of use and a claim of low polymerization shrinkage compared with conventional composites.^9^ These materials have a short curing time due to new initiation systems and increased translucency based on reduced filler amounts and increased filler size.^10^ Furthermore, polymerization shrinkage stresses are reduced through the incorporation of stress-relievers; thus, they have a decreased risk of gap formation at the tooth-restoration interface.^11^

Gaps on the margins of the restorations may cause material deterioration and marginal infiltration.^12^ Although bulk-fill composite resins are claimed to exhibit low polymerization shrinkage, there is not enough information with respect to the effects of gap formation of bulk-fill composites using an intermediate liner in the literature. The use of a liner (flowable composites, resin-modified glass-ionomers, filled adhesives) with a low elastic modulus/low viscosity could provide better cavity adaptation with less gap formation as a stress-absorbing layer and lessen the polymerization shrinkage at the tooth-restoration interface.^13^

Currently, different types of bulk-fill composite resins that are classified according to their rheological properties are commercially available.^14^ For this *in vitro* study, high-viscosity, sonic-activated and low-viscosity bulk-fill composite resins were used. The purpose of this *in vitro* study was to assess the gap formation volume of maxillary premolars restored with three different types of bulk-fill resin composites, with and without a resin-modified glass-ionomer cement liner (RMGIC) as an intermediate material using micro-computed tomography.

The research null hypotheses were:

There would be no difference in the gap formation volume between different types of bulk-fill composite resins.

The RMGIC liner would not reduce gap formation volume and enhance the cavity adaptation of teeth restored with bulk-fill composite resins.

## Methodology

This *in vitro* study was approved by the local ethics committee (process no. 06/06/2018-9063).

### Sample Size Calculation

The sample size was calculated based on the estimated effect size between groups according to the literature.^15^^,^^16^ It was determined that 10 samples were needed for each group to achieve a medium effect size (d=0.50), with 80% power and a 5% type 1 error rate in this study.

### Specimen Preparation

A total of 60 intact human maxillary premolar teeth, freshly extracted for orthodontic and periodontal purposes, were selected. To standardize the dimensions of the teeth before the study, the maximum bucco-palatal width (BPW) of each tooth was measured using a digital micrometer.^17^ Then, the teeth were allocated into six groups according to the BPW (n=10). The mean bucco-palatal dimensions of the teeth between groups differed no more than 5% (p=0.061) according to one-way ANOVA using the Statistical Package for Social Sciences 22.0 for Windows software (SPSS 22.0 for Windows, SPSS Inc., Chicago, IL, US) (p<0.05).

The teeth were embedded in acrylic resin blocks with the crown extended to 2 mm from the cementoenamel junction (CEJ) along the vertical axis.

**Figure 1:**
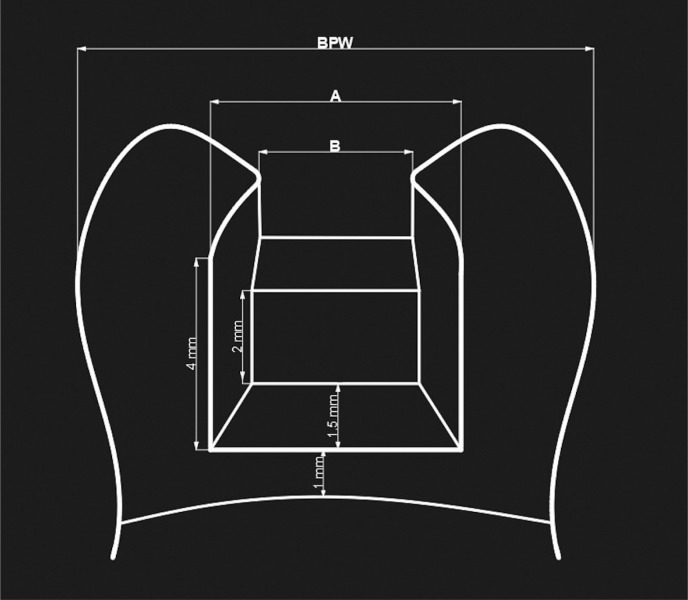
Schematic diagram of the MOD cavity design. Bucco-palatal width (BPW), gingival floor width (A= 2/3 BPW), occlusal isthmus width (B=1/2 BPW)

A standardized Class II mesio-occluso-distal (MOD) cavity was opened in each tooth (Figure 1) using a coarse diamond fissure bur (FC Diamond, GZ Instrumente, Austria) in a high-speed handpiece under water cooling. A new bur was employed for each of the five specimens. The dimensions of the approximal box of each cavity were arranged such that they were two-thirds of the BPW of the tooth (A), and the occlusal isthmus was arranged to half of the BPW (B). The total depth of the cavity was adjusted to 4 mm, with an axial wall height of 2 mm. Approximal boxes had 1.5 mm mesiodistal width on the gingival floors 1 mm above the CEJ.^18^ The dimensions of cavity preparation were confirmed with a digital caliper. The specimens were then stored in distilled water at room temperature (23±1°C) before and after preparation.

### Restorative Procedure

**Figure 2:**
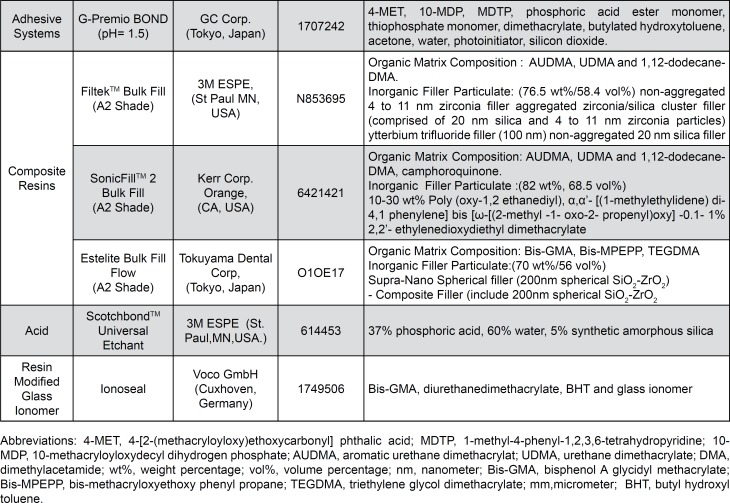
Material brand names/ manufacturers, batch numbers and chemical compositions

After cavity preparations, a metal auto matrix (SuperMat™ assorted kit, Kerr Corp., Orange, USA) was placed around the tooth. The enamel margins of the cavities were etched with 37% phosphoric acid for 15 s, rinsed with water for 5 s and gently air-dried. Then, a single-component universal adhesive system, G-Premio Bond (GC Corp., Japan) was applied with a microbrush for 10 s, followed by air-thinning for 5 s under maximum air pressure and curing with a light-emitting diode light curing unit (LED LCU) (Valo, Ultradent, South Jordan, UT, USA) (irradiance of 1000 mW/cm^2^). The light intensity was controlled during the whole process using a radiometer (Demetron LED Radiometer, Kerr Corp.). The materials used in this study are summarized in Figure 2.

#### Group Filtek Bulk Fill (FB)

High-viscosity bulk-fill resin composite (Filtek™ Bulk Fill, A2 Shade, 3M ESPE, USA) was used to restore the cavity and was polymerized with an LED LCU from the occlusal surface for 10 s. After removing the metal matrix, the restorations were polymerized from the mesial and distal surfaces for 10 s on each side.

#### Group Filtek Bulk Fill with liner (FBL)

The cavities were lined with one-component RMGIC (Ionoseal, Voco GmbH, Germany) liner, approximately 1 mm thick, on the pulpal and axial walls and light-cured with an LED LCU for 20 s. G-Premio Bond was applied in the selective-etch mode as previously described, and the cavity was restored with Filtek™ Bulk Fill resin composite as described for group FB.

#### Group SonicFill 2 (SF)

Sonic-activated high-viscosity bulk-fill resin composite (SonicFill™ 2, A2 Shade, Kerr Corp.) was used to restore the cavity with a sonic hand-piece and polymerized with an LED LCU for 20 s. After removing the metal matrix, the restorations were polymerized from the mesial and distal surfaces for 10 s on each side.

#### Group SonicFill 2 with liner (SFL)

The cavities were lined with RMGIC liner, polymerized as described in group FBL. G-Premio Bond was applied as previously described and then restored with SonicFill™ 2 resin composite as described in group SF.

#### Group Estelite Bulk Fill Flow (EB)

Low-viscosity bulk-fill resin composite (Estelite Bulk Fill Flow, A2 Shade, Tokuyama Dental Corp., Japan) was used to restore the cavity, and it was polymerized with an LED LCU for 10 s. After removing the metal matrix, the restorations were polymerized from the mesial and distal surfaces for 10 s on each side.

#### Group Estelite Bulk Fill Flow with liner (EBL)

The cavities were lined with a RMGIC liner and polymerized as described for group FBL. G-Premio Bond was applied as previously described, followed by restoration with Estelite Bulk Fill Flow as described for group EB.

All restorations were finished with an extra-fine diamond bur (FC Diamond, GZ Instrumente, Austria) with a high-speed handpiece under water cooling and polished with aluminum oxide polishing disks (Sof-Lex, 3M ESPE, USA) in a slow hand-piece according to the manufacturer's instructions.

### Aging Procedure

All restored teeth were thermocycled (SD Mechatronik Termocycler THE-1100, Feldkirchen-Westerham, Germany) for 10,000 cycles between 5°C and 55°C with a dwell time of 30 s and a transfer time of 10 s. Then, the specimens were fixed to a chewing simulator (CS-4.2; SD Mechatronik, Feldkirchen-Westerham, Germany) and subjected to 50,000 cycles (100 N and 1.7 Hz) at room temperature (23±1°C) and 100% humidity. A vertical load was applied with a 3.2-mm stainless-steel ball-shaped stylus at the center of the restorations.^19^ During the aging procedure, the specimens remained immersed in distilled water.

### Micro-Computed Tomography (micro-CT) Analysis

Gap formation analysis was performed with the microtomography system SkyScan 1174v2 (Skyscan, Kartuizersweg, Kontich, Antwerp, Belgium). The micro-focus X-ray source was set at 50-kVp accelerating voltage, 40 W and 800 μA beam current, and a 0.5 mm aluminum filter was applied. The specimens were scanned at a 14.46 μm pixel size at 1024x1304 resolution with an exposure time of 7500 ms. The total number of slices averaged 360, with an average scanning time close to 50 min. For each sample, 360 raw data points were recorded, and, after reconstruction, 655 transverse tomographic sections were obtained using NRecon (Version 1.6.10.2, Skyscan, Kontich) software.

Image analyses of gap formation based on the volume of black spaces were conducted with the three-dimensional (3D) analysis tool from CTAn (CT-Analyser software Version 1.16.4.1; Skyscan, Kontich). Black spaces were present in the volume of interest (VOI), which originated from whole two-dimensional (2D) images within the region of interest (ROI). All evaluations were performed with the VOI achieved from the ROI centered on the delimitations of the restorative materials. 3D images were obtained by CTvox (Version 3.1.1 r1191, Skyscan, Kontich).^15^ The volume of gap formation was calculated through analysis of the tooth-restoration interface and is described in mm^3^.

### Statistical Analysis

Statistical analysis was performed using SPSS 22.0 for Windows (SPSS Inc., Chicago, IL, USA). The gap formation data were first analyzed for normality of variables with the Shapiro-Wilk test, and Levene's test was used to show homogeneity of variances. These data were normally distributed. Repeated-measures ANOVA was used to compare within-and between-group differences in gap formation. Pairwise comparisons were performed with Bonferroni correction. Statistical significance was determined at a confidence level of 0.05 in all analyses.

## Results

**Figure 3:**
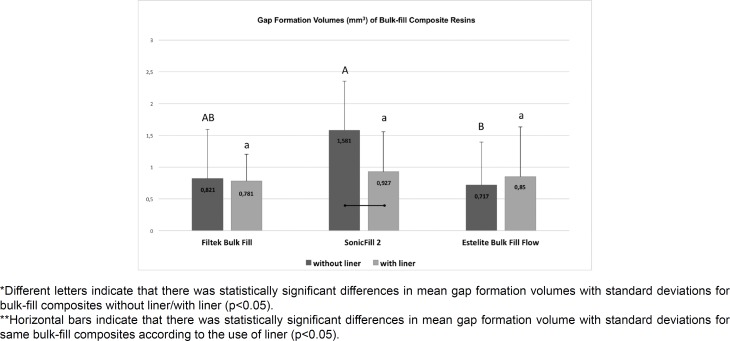
Micro-CT-based gap formation volumes with standard deviations (mm^3^) of all tested groups (n=10)

**Figure 4:**
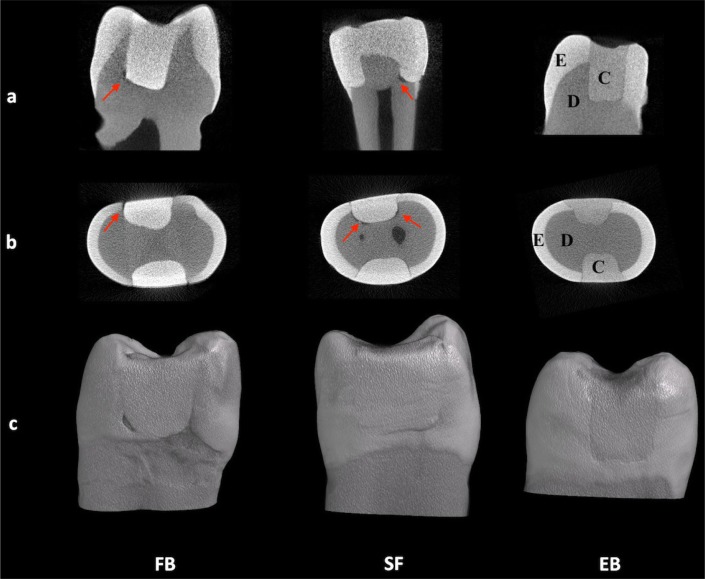
Representative two-dimensional (2D) and three-dimensional (3D) images of all tested bulk-fill composites by micro-CT. The gap formations are detected between teeth and restorations (arrows). Illustrative 2D images of the specimens are visualized: sagittal section (a) and axial section (b). 3D volume rendering of the specimens (c). E, enamel; D, dentin; C, composite; FB, Filtek Bulk Fill; SF, SonicFill 2; EB, Estelite Bulk Fill Flow

The obtained data were assessed based on the recorded volume (mm^3^). An analysis of the gap formation between bulk-fill composites and/or the RMGIC liner and cavity walls was performed for all tested groups (n=10). Micro-CT-based gap formation volumes with standard deviations are shown in Figure 3. The representative two-dimensional (2D) and three-dimensional (3D) images of all tested bulk-fill composites by micro-computed tomography (micro-CT) are shown in Figure 4. Group SF showed a significantly higher gap formation volume than Group EB (p<0.05). There were no statistically significant differences between Group EB and Group FB (p>0.05).

**Figure 5:**
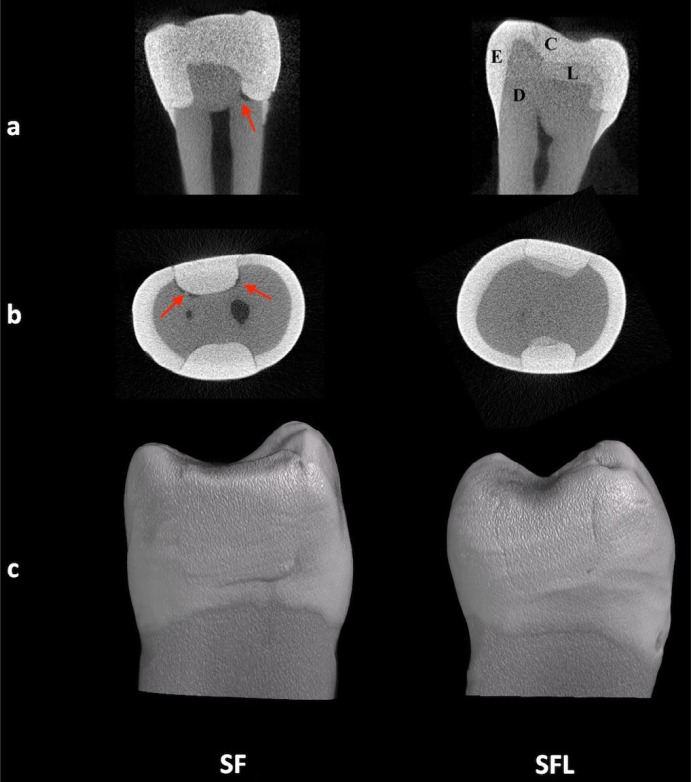
Representative two-dimensional (2D) and three-dimensional (3D) images of sonic-activated bulk-fill composites by micro-CT. Presence of gap formation is noted at the tooth-restoration interface (arrows). Illustrative 2D images of the specimens are visualized: sagittal section (a) and axial section (b). 3D volume rendering of the specimens (c). E, enamel; D, dentin; C, composite; SF, SonicFill 2; SFL, SonicFill 2 with liner

In addition, within the groups with a liner, no significant differences were found between Group FBL, Group SFL and Group EBL (p>0.05). When comparing the groups restored with the same bulk-fill composites regarding the use of a liner, Group SFL showed significantly lower gap formation volumes than Group SF (Figure 5) (p<0.05). There were no statistically significant differences between Group FB and Group FBL or Group EB and Group EBL (p>0.05).

## Discussion

The gap formation volume of teeth restored with different types of bulk-fill composites, with and without a RMGIC liner, was evaluated. Based on the results of this study, the first null hypothesis, which proposed that there would be no difference in the gap formation volume between different types of bulk-fill composites, was rejected. The low-viscosity bulk-fill composites exhibited lower gap formation volumes than the other tested bulk-fill composites. The second null hypothesis, which proposed that a RMGIC liner would reduce gap formation volume and enhance the cavity adaptation of teeth restored with bulk-fill composites, was partially rejected. When an RMGIC liner was used under all tested bulk-fill composites, a significant reduction in gap formation was identified for sonic-activated bulk-fill composites.

Gap formation is one of the most common issues associated with composite resin restorations.^20^ Gaps can originate from various factors, including inadequate adhesion at the tooth-restoration interface due to polymerization shrinkage, adhesive resin degradation with insufficient light-curing, fatigue resulting from the aging procedure, differences in the coefficients of thermal expansion of the tooth substrate and composite resin, the finishing and polishing procedure, and lack of restorative material placement in the cavity.^21^

Several *in vitro* methods are available to assess the gap formation of restorations, such as dye penetration, air pressure, fluid filtration, optical coherence tomography (OCT) and X-ray micro-computed tomography (micro-CT).^22^ Conventional methods (i.e., dye penetration) are destructive due to the need to section the specimens and are semi-quantitative, based on visual evaluations by the operator. Furthermore, they do not represent the entire gap formation areas.^23^ To overcome these drawbacks, a novel methodology, micro-CT, has been introduced as an imaging device, with its origins in the further development of conventional computed tomography.^24^ This device can provide 2D and 3D images of gap/void formation in restored teeth due to the penetrating capacity of X-rays^24^ and is a powerful method for ensuring the acquisition of precise information that would allow clinicians to analyze the area without destroying the specimens.^25^

In this study, micro-CT imaging was used to quantify the gap formation between the cavity walls and restorative materials as the volume (mm^3^) after a thermo-mechanical aging procedure. Thermocycling and mechanical aging are the most effective and frequently used methods for imitating clinical situations.^26^ Thermocycling is a water storage protocol that subjects specimens to the extreme temperature differences present in the oral cavity due to hot or cold drinks, inducing the composite resin to contract and expand several times for hydrolytic degradation.^27^ Mechanical aging is performed to simulate the exposure of the tooth-restoration interface to cyclic subcritical loadings produced during chewing.^28^ In the current study, all restored teeth were subjected to 10,000 thermocycles (5-55°C), which represents 1 year of clinical functions,^29^ and 50,000 simulated chewing cycles (100 N loading).

Significantly higher gap formation volumes were found for Group SF compared with Group EB among all tested bulk-fill composites. In contrast to this finding, Han, et al.^16^ (2017) reported that low-viscosity bulk-fill composites showed a higher gap formation volume compared with sonic-activated and high-viscosity bulk-fill composites. Additionally, Jung and Park^30^ (2017) and Hayashi, et al.^31^ (2019) reported that high-viscosity bulk-fill composites showed better marginal adaptation than low-viscosity bulk-fill composites. Alqudaihi, et al.^32^ (2019) stated that no significant differences were found between different types of bulk-fill composites with respect to cavity adaptation. Estelite Bulk Fill Flow has lower filler content (70 wt%/56 vol%) associated with the high percentage of organic matrix compared with Filtek Bulk Fill (76.5 wt%/ 58.4 vol%) and SonicFill™ 2 (82 wt%/68.5 vol%). Furthermore, it is well known that flowable composite resins may provide better adaptation to the cavity walls due to their lower viscosity.^33^ Therefore, this finding can be explained by the lower filler content of this material and new filler technology (spherical filler) present in its inorganic matrix. SonicFill™ 2 Bulk Fill utilizes sonic energy with a blend of low-viscosity composite and universal composite. It is composed of high filler content and different monomers (AUDMA, UDMA) that decrease the polymerization shrinkage of the material. In addition, it can provide better adaptation to cavity walls, behaving like a flowable composite during placement.^34^ However, a few studies^35^^,^^36^ have reported that of the previous generation of sonic-activated bulk-fill composites, SonicFill™ did not provide better adaptation to cavity walls compared with conventional composites. In this study, despite the use of a new-generation sonic-activated bulk-fill composite with a new filler technology (zirconium oxide and silica oxide particles), similar gap formation volumes to those in other studies^35^^,^^36^ were obtained in Group SF. Moreover, this finding can be explained by the long-term thermocycling, in addition to mechanical aging, unlike in previous studies.^35^^,^^36^ As a consequence, the aging procedure resulted in deterioration at the tooth-restoration interface.

An intermediate liner application with a low elastic modulus has been recommended to reduce polymerization shrinkage as well as gap formation of composite resins.^37^ Nie, Yap, Wang^38^ (2018) reported that reduced gap formation was observed when an intermediate liner was used under conventional composite resins, based on the improved cavity adaptation and stress absorbing capacity. Nevertheless, Alomari, Reinhardt, Boyer^39^ (2001) determined no differences in gap formation between the restorations with and without a liner. Although manufacturers claim that bulk-fill composites show lower polymerization shrinkage than conventional composites, there is not enough information in the literature regarding gap formation of bulk-fill composites when intermediate liners are used.

In the present study, gap formations of different bulk-fill composites with and without a resin-modified glass-ionomer cement (RMGIC) liner were evaluated. Among all the tested groups, the RMGIC liner produced a significant reduction in gap formation only in group SFL compared with group SF. Han, et. al.^40^ (2019) reported that cavity adaptation increased when an intermediate liner (Fuji Lining LC, GC Corp.) was used under high-viscosity bulk-fill composite resin (Tetric EvoCeram Bulk Fill, Ivoclar Vivadent). This finding can be explained by the different chemical composition of the materials used in both studies and cavity configuration. In addition, it was determined that SonicFill™ 2 Bulk Fill showed the highest gap formation volume among all the tested bulk-fill composites in this study. Thus, the RMGIC liner, due to its low elastic modulus, significantly decreased gap formation and facilitated cavity adaptation of this material. As a consequence, the use of an RMGIC liner could be recommended when sonic-activated bulk-fill composites are utilized.

## Conclusions

Regarding the limitations of this study, only one type of intermediate liner (RMGIC) was investigated to evaluate the effects on gap formation under bulk-fill resin composite restorations. In addition, a conventional composite used as a control group was needed for comparison with the bulk-fill composites. Thus, further studies should focus on the effects of different intermediate liners, such as the RMGIC liner (Vitrebond, 3M ESPE) or flowable resin composite, under both bulk-fill and conventional composite restorations.

Within the limitations of the present study, it can be concluded that:

Different types of bulk-fill composites affected the gap formation volume. Low-viscosity bulk-fill composites showed better cavity adaptation and less gap formation than sonic-activated bulk-fill composites.

The use of an RMGIC liner yielded a significant reduction in gap formation volume for only sonic-activated bulk-fill composites.
